# Facts for the People

**Published:** 1882-09

**Authors:** 


### FACTS FOR THE NATIVES.

It is a matter of envious remark, and ought to be one of grave reflection, that foreigners "succeed" better than Americans. In our cities and large towns, both on the seaboard and on the great rivers of the interior, the rule is, that foreigners are going up, and that native citizens are going down, as to the extent of their business, their pecuniary resources, their social position, and influence. In Cincinnati and St. Louis, it is the Dutchman who owns the corner buildings. In New York City, it is the Dutchman who is able to rent or own the corner groceries. Germans and Englishmen here wield the most capital. Our heaviest importers, our very largest commission-houses, have Europeans at their head. We can count in this city alone, foreigners by the dozen, Scotchmen, Irishmen, Germans, English, and others, who, in their youth, were without a dollar, and thousands of miles from home and friends and kindred, and, at the end of thirty years, are to-day worth their hundreds of thousands, and some estimate their fortune at millions.

On the other hand, there are multitudes of the children of native-born citizens who had fortunes to begin with, but who are now bankrupt in money, in character, and in influence; and, thriftless and idle, are going down to an early, or besotted grave.

What makes this wide difference? The poor foreigner comes here with a vivid sense of the evils and the degradation of poverty; he feels that money gives power and influence and position; and, being free to make it, he is willing to work and to save; he is industrious, self-denying and frugal, and the result is, that he rises from the very first hour that his foot touches Castle Garden. It matters not what his position was at home, he embraces the very first opportunity of earning a penny; and, if he cannot get wages at first, he will work for his board, until he can look around, or make his employer feel his worth.

**BUCKWHEAT-CAKES.**—Take about two quarts of water and one pint of milk, mixing in the buckwheat-meal, and about half a pint of brown flour, (the "middlings" of wheat.) This we think, makes them much better than all buckwheat. Stir in two table-spoonfuls of salt.

